# Optimization of concentrations and exposure durations of commonly used positive controls in the in vitro alkaline comet assay

**DOI:** 10.1093/toxres/tfae195

**Published:** 2024-12-04

**Authors:** Seda İpek Tekneci, Aylin Üstündağ, Yalçın Duydu

**Affiliations:** Ankara University, Faculty of Pharmacy, Department of Pharmaceutical Toxicology, 06560, Ankara, Türkiye; Ankara University, Graduate School of Health Sciences, 06110, Ankara, Türkiye; Ankara University, Faculty of Pharmacy, Department of Pharmaceutical Toxicology, 06560, Ankara, Türkiye; Ankara University, Faculty of Pharmacy, Department of Pharmaceutical Toxicology, 06560, Ankara, Türkiye

**Keywords:** Comet assay, Positive controls, *in vitro*, Standardization

## Abstract

Endogenous and exogenous factors cause DNA damage through chemical changes in the genomic DNA structure. The comet assay is a versatile, rapid, and sensitive method for evaluating DNA integrity at the individual cell level. It is used in human biomonitoring studies, the identification of DNA lesions, and the measurement of DNA repair capacity. Despite its widespread application, variations between studies remain problematic, often due to the lack of a common protocol and appropriate test controls. Using positive controls is essential to assess inter-experimental variability and ensure reliable results. Hydrogen peroxide (H_2_O_2_) is the most commonly used positive control, while potassium bromate (KBrO₃), methyl methanesulfonate (MMS), ethyl methanesulfonate (EMS), *N*-ethyl-*N*-nitrosourea (ENU), and etoposide are used less frequently. However, differences in concentrations and exposure durations prevent the confirmation of test method efficacy. This study investigates the dose–response relationship for H_2_O_2_, KBrO_3_, MMS, EMS, ENU and etoposide in the comet assay for 30 and 60-minute exposure durations in 3T3 cell lines. Accordingly recommended concentrations and exposure durations were found to be 50 μM 30 minutes (H_2_O_2_); 500 μM 60 min. (MMS); 10 μM 30 min. (Etoposide); 0.2 mM 30 min. and 2 mM 60 min. (EMS); 2 mM 30 min. (ENU); 500 μM 30 min. and 50 μM 60 min. (KBrO_3_). Our findings will contribute to reducing inter-laboratory variability by offering guidance on selecting doses and exposure durations for positive controls in the *in vitro* alkaline comet assay.

## Introduction

DNA damage from exposure to endogenous or exogenous factors is a term that includes chemical changes that occur in the genomic structure of DNA.^1–3^ One of the widely employed genotoxicity tests that evaluate the integrity of DNA is the comet assay.^1^^,^^3^ The comet assay is a versatile, rapid, and sensitive technique that assesses the DNA integrity at the individual cell level.^2^^,^^4^ It has wide application areas and can be integrated into various fields, such as human biomonitoring studies. It can also be utilized to identify DNA lesions by using diverse enzyme modifications and to measure DNA repair capacity.^1^^,^^4^^,^^5^

The chemical compound or material used to confirm whether a test method operates as intended is commonly known as the positive control.^6^^,^^7^ Positive controls are selected based on the expected outcomes for the study and are essential in vitro genotoxicity tests to provide crucial information about the experiment’s performance.^8^ Additionally, positive controls utilized in the comet assay are crucial for assessing inter-experimental variabilities and ensuring result reliability.

Although the comet assay is one of the most widely used genotoxicity tests,^9–12^ there are variations in interindividual and interlaboratory studies.^9^^,^^12^ Theoretically, if the test conditions are the same, the damage to DNA is proportional to the level of DNA migration due to electrophoresis application. However, the fact that DNA damage levels are not the same in different laboratories for the same chemical has become an important problem of the in vitro comet assay. The main reason for these differences is the lack of a common protocol for optimum test conditions and appropriate test controls.^11^ These differences have been demonstrated by studies involving different laboratories since the early 2,000s.^10^ For example, the “European Standards Committee on Oxidative DNA damage (ESCODD)” reported that there were differences in the formamidopyrimidine DNA glycosylase (Fpg)-sensitive regions of the same cells in different laboratories.^11^

Differences in the interpretation of the results obtained with the comet assay are a factor limiting the potential of the method.^9^ To date, various committees and groups hCOMET, European Comet Assay Validation Group (ECVAG), European Standards Committee on Oxidative DNA damage (ESCODD) have carried out studies to reduce interlaboratory variation.^9^^,^^11^^,^^12^ For example, standardization of positive controls is important for accurate interpretation of test results. In this context, hCOMET partners reported that potassium bromate (KBrO_3_) is a suitable positive control for the Fpg-modified comet assay.^11^

When reviewing the literature on in vitro comet assay, H_2_O_2_ emerged as the most commonly utilized positive control. Moreover, KBrO_3_, MMS, EMS, ENU, and etoposide have also been consistently used as positive controls in the comet assay, although at a lower frequency. However, using different concentrations and exposure durations for positive controls by various study groups makes it challenging to confirm whether the test method operates as intended.

In this study, we aimed to investigate the dose–response relationship for the most commonly used positive controls (H_2_O_2_, KBrO_3_, MMS, EMS, ENU, etoposide) in comet assay for two exposure durations (30 and 60 min) in mouse embryo fibroblast (3T3) cell lines. In this way, we aimed to contribute to research focused on reducing inter-laboratory variabilities in comet assay outcomes. This study will provide valuable guidance for selecting doses and exposure durations of positive controls when conducting an in vitro alkaline comet assay.

## Materials and methods

### Cell line and culture conditions

3T3 (Swiss albino) cell lines of mouse embryonic fibroblasts were used in this study (ATCC® CCL-92, Germany). In the present study, the 3T3 cell line was selected for use, as it has been employed in a number of previous studies. Moreover, there is no existing literature utilizing 3T3 cell lines for the optimization of positive controls in the in vitro alkaline comet assay.

The cells were cultured in DMEM, supplemented with 10% FBS and 1% penicillin/streptomycin under 5% CO_2_ and 37°C. Once the cells had reached 70%–80% confluency, they were passaged. The cells were detached using a 0.25% trypsin/EDTA solution. The viability and number of cells were determined using the trypan blue dye exclusion method. A seeding density of 5-6 × 10^5^ cells was employed in T25 flasks for subsequent passages and the assay.

## Reagents

H_2_O_2_, MMS, EMS, ENU, KBrO_3_, normal and low melting point agarose, %0.25 trypsin–EDTA, Penicillin/Streptomycin (Pen/Strep), Dimethyl sulfoxide (DMSO), ethidium bromide were purchased from Sigma-Aldrich, Germany. Etoposide was obtained from Cayman Chemical, USA. Dulbecco’s Modified Eagle’s Medium (DMEM) and trypsin were sourced from Sartorius, Israel. Fetal bovine serum (FBS) (heat-inactivated) was purchased from Biological Industries, Israel. Chemicals such as hydrochloric acid (HCl), sodium hydroxide (NaOH), tris, and triton X-100 were acquired from Merck, Germany. Disodium ethylenediaminetetraacetic acid (Na_2_EDTA), sodium chloride, and sodium lauryl sarcosinate were obtained from Amresco, USA.

## COMET assay

The alkaline comet assay was conducted using 3T3 cell lines according to the standard protocol established by Singh et al. (1988).^13^

Briefly, the cells were seeded into six-well plates with 150.000 cells/ 2 mL DMEM in each well. H_2_O_2_ (1, 5, 10, 25, 50, 75, 100, and 200 μM), MMS (1, 5, 10, 50, 100, and 500 μM), EMS (0.05, 0.1, 0.2, 0.5, 1, and 2 mM), ENU (0.025, 0.05, 0.01, 0.2, 0.4, and 2 mM), etoposide (0.05, 0.1, 0.5, 1, 5, and 10 μM) and KBrO_3_ (50, 100, 500, 1000, 2500, and 5000 μM) were the concentrations of positive controls we intended to study. H_2_O_2_ and KBrO_3_ solutions were prepared in sterile distilled water, while the others were dissolved in sterile DMSO. The dose response of these positive controls was investigated in two different durations of exposure (30 and 60 min). After harvesting the cells, 100 μl low-melting point agarose (0.5% LMPA) was mixed with 50 μl of cell suspension (1-2 × 10^4^ cells/slide) at 37 °C. Thereafter, cell suspensions were embedded into the precoated slides with 1% NMPA, and the slides were left to enable the agarose to solidify on an ice-cold tray for 5 min. After solidification, the slides were soaked into a cold lysing solution (10 mM Tris, 2.5 M NaCl, 100 mM Na_2_EDTA, 1% sodium sarcosinate, 1% Triton-X 100, 10% DMSO at pH = 10.0) prepared before for at least 1 h at 4 °C. The tank was filled with a cold electrophoresis solution (1 mM Na_2_EDTA, 300 mM NaOH, at pH = 13). At the end of the lysing time, the slides were washed with distilled water at once, and placed into the electrophoresis tank. The electrophoresis was performed for 20 minutes at 300 mA and 0.7 V/cm after 20 minutes for the alkali denaturation. The slides to be removed from the tank were washed off with distilled water for five minutes and with a neutralizing solution (0.4 M Tris at pH = 7.5) for 15 minutes. Fixation with ethyl alcohol procedure was applied to the cells, and stored at room temperature until the counting process under the microscope. To examine the cells under a microscope (Leica DM fluorescent microscope, Germany), slides were stained with 60 μL (20 μg/mL) ethidium bromide solution. 100 randomly chosen cells were counted by using the COMET Assay IV Software program (Instem, UK). The DNA damage was indicated as a percentage of mean tail intensity.

### Statistical analysis

Statistical analysis was performed using SPSS software (Version 23.0, SPSS Inc., USA). The results were expressed as mean ± standard error of the mean (SEM). One-way analysis of variance (ANOVA) was conducted to analyze all chemical groups and exposure times. Post-hoc analysis, specifically Fisher’s least significant difference test (LSD), was employed for between-group comparisons. A significance level of *P* < 0.05 was considered statistically significant.

### Trypan blue assay

A 20 μL sample of the cell suspension was diluted with an equal volume of trypan blue. The cell counting slide was covered with a 10 μL aliquot of this cell suspension. The total and viable cell count and percentage viability in 1 mL were determined using the cell counter (Bio-Rad TC20, Singapur).

## Results

**Fig. 1:**
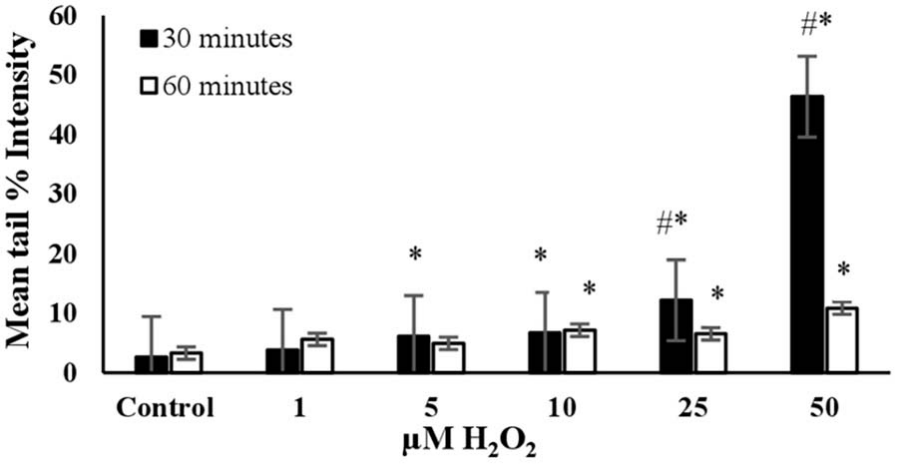
The mean tail % intensity (*n* = 100) ± SEM in 3T3 cells following exposures to H_2_O_2_ for 30 and 60 min. ^*^Statistically significant between control and exposed cells (*p* < 0.05). ^#^Statistically significant between 30 and 60 min of exposure duration (*p* < 0.05).

**Fig. 2:**
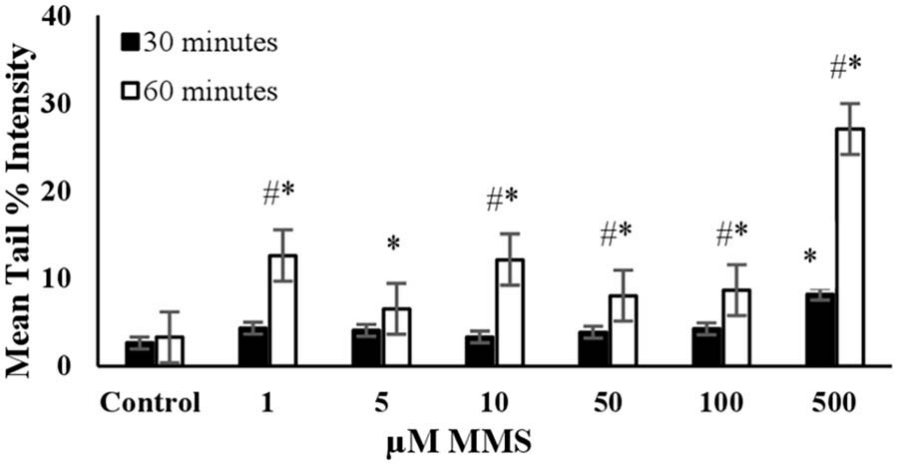
The mean tail % intensity (*n* = 100) ± SEM in 3T3 cells following exposures to MMS for 30 and 60 min. ^*^Statistically significant between control and exposed cells (*p* < 0.05). ^#^Statistically significant between 30 and 60 min of exposure duration (*p* < 0.05).

**Fig. 3:**
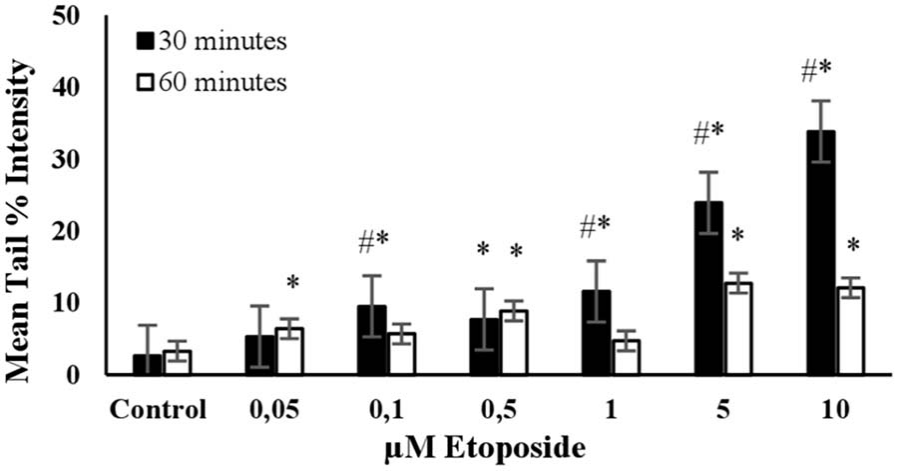
The mean tail % intensity (*n* = 100) + SEM in 3T3 cells following exposures to etoposide for 30 and 60 min. ^*^Statistically significant between control and exposed cells (*p* < 0.05). ^#^Statistically significant between 30 and 60 min of exposure duration (*p* < 0.05).

**Fig. 4:**
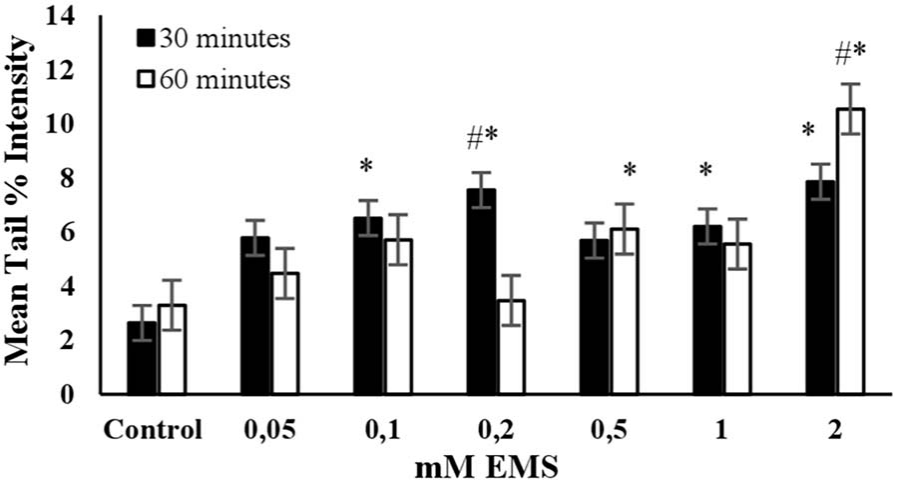
The mean tail % intensity (*n* = 100) ± SEM in 3T3 cells following exposures to EMS for 30 and 60 min. ^*^Statistically significant between control and exposed cells (*p* < 0.05). ^#^Statistically significant between 30 and 60 min of exposure duration (*p* < 0.05).

**Fig. 5:**
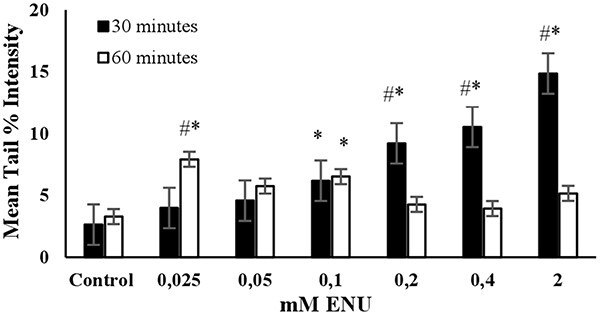
The mean tail % intensity (*n* = 100) ± SEM in 3T3 cells following exposures to ENU for 30 and 60 min. ^*^Statistically significant between control and exposed cells (*p* < 0.05). ^#^Statistically significant between 30 and 60 min of exposure duration (*p* < 0.05).

**Fig. 6:**
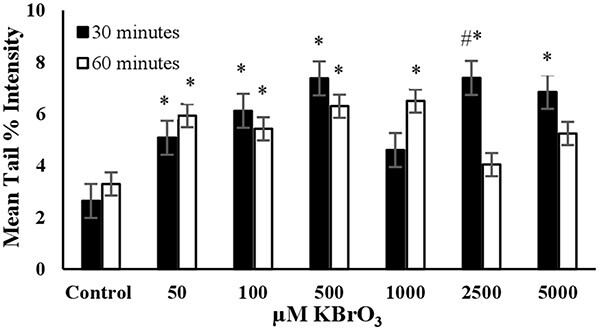
The mean tail % intensity (*n*=100) ± SEM In 3T3 cells following exposures to KBrO_3_ for 30 and 60 min. ^*^Statistically significant between control and exposed cells (*p* < 0.05). ^#^Statistically significant between 30 and 60 min of exposure duration (*p* < 0.05).

The DNA damage in a randomly selected group of 3T3 cells (*n* = 100) was measured as the mean tail % intensity using COMET Assay IV Software. All positive controls were able to induce DNA damage after reaching a certain concentration, as shown in Figs 1–6.

**Fig. 7:**
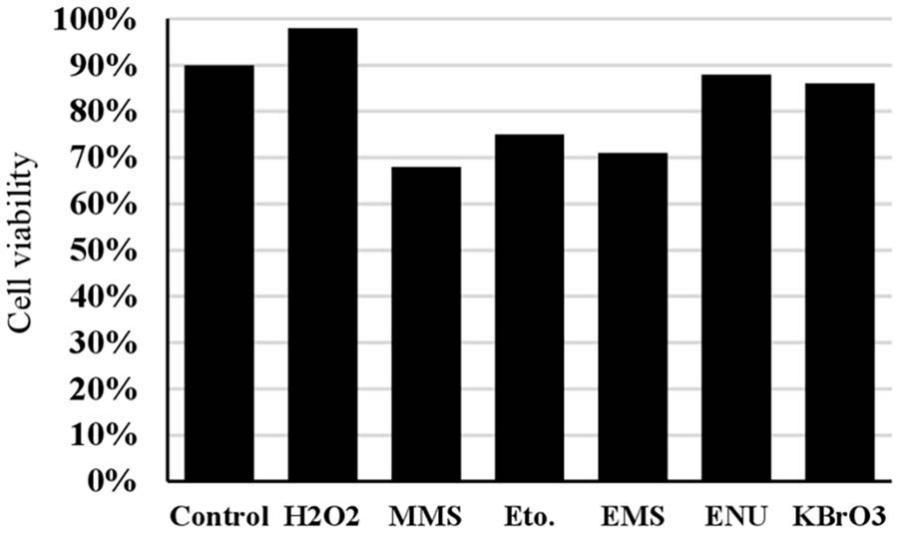
The cell viability for all positive controls at the concentrations and exposure duration given in Table 1 (Eto.: Etoposide).

The concentrations and exposure durations used in the trypan blue assay (cell viability) for positive controls are detailed in Table 1. The viabilities of 3T3 cells for all positive controls at the specified concentrations and exposure durations listed in Table 1 are shown in Fig. 7.

**H_2_O_2_:** The mean tail % intensity, which represents DNA damage, increased in a dose-dependent manner when cells were exposed to concentrations ranging from 1 to 50 μM for 30 minutes (Fig. 1). Higher concentrations (75, 100, 200 μM) were also tested. At these higher concentrations, complete migration of the DNA from the nucleus into the tail was observed, resulting in what are termed “ghost cells (clouds)”.^14^ Therefore, these concentrations were not included in Fig. 1. The mean tail intensity for the concentrations of 25 μM and 50 μM was significantly lower at 60 min of exposure compared to 30 min, possibly due to the initiation of DNA repair. The cell viability (Trypan Blue Assay) was found to be higher than 90% when exposed to 50 μM H_2_O_2_ for 30 min (see Fig. 7). This condition resulted in a noticeable tail under the fluorescent microscope, with the mean tail % intensity being higher than 40 (see Fig. 1). Therefore, it was concluded that the optimal conditions for using H_2_O_2_ as the positive control in the alkaline comet assay were a 30-min exposure duration and a concentration of 50 μM (Table 2).

**Table 1:** The cell viability at concentrations and exposure durations of positive controls.

Positive controls	Concentration	Exposure duration
H_2_O_2_	50 μM	30 min
MMS	500 μM	60 min
Etoposide	10 μM	30 min
EMS	2 mM	60 min
ENU	2 mM	30 min
KBrO_3_	2.5 mM	30 min

**Table 2:** Recommended concentrations and exposure durations for the positive controls used in alkaline comet assay.

Positive controls	Recommended concentrations	Recommended exposure durations	Corresponding mean tail % intensities
H_2_O_2_	50 μM	30 minutes	46.35
MMS	500 μM	60 minutes	27.04
Etoposide	10 μM	30 minutes	33.85
EMS	0.2 mM 2 mM	30 minutes 60 minutes	7.54 10.54
ENU	2 mM	30 minutes	14.86
KBrO_3_	500 μM 50 μM	30 minutes 60 minutes	7.37 5.93

**MMS:** The DNA damage was significantly higher than the control in all tested concentrations after 60 min of exposure, as shown in Fig. 2. However, the mean tail % intensities for concentrations ranging from 1 to 100 μM were not statistically different from the control after 30 min of exposure. This indicates that short-term exposure (30 min) did not lead to a significant increase in mean tail % intensities for MMS. The increase in mean tail % intensity was significant (*P* < 0.05) for 500 μM MMS after 60 min of exposure. Under these conditions, cell viability was slightly lower than 70% (Fig. 7). Therefore, 60 min of exposure and 500 μM concentration were determined to be the optimal conditions for using MMS as the positive control in the alkaline comet assay (Table 2).

**ETOPOSIDE:** The level of DNA damage in cells increased with the dose when exposed to concentrations ranging from 0.05 to 10 μM for 30 min (Fig. 3). There was no significant increase in mean tail % intensities with concentrations between 0.05 and 1 μM after 60 min of exposure. The increase in average tail % intensities was only significant for 5 and 10 μM concentrations of etoposide after a 60 min exposure. However, a noticeable increase in mean tail % intensity was observed for 10 μM etoposide after a 30- min exposure. Under these experimental conditions, cell viability was higher than 70% (Fig. 7). Accordingly, a 30 min exposure and a 10 μM concentration were determined as the optimal conditions for using etoposide as the positive control in the alkaline comet assay (Table 2).

**EMS:** The highest mean tail % intensity was observed with a 2 mM EMS concentration during a 60-min exposure, with statistical significance (*P* < 0.05). Additionally, a linear increase in mean tail % intensity was observed between concentrations of 0.05 and 0.2 mM EMS during a 30- min exposure (see Fig. 4). Additionally, equal mean % tail intensity values were detected for 0.2 mM and 2 mM EMS concentrations. The cell viability was slightly higher than 70% for 2 mM EMS at an exposure duration of 60 min (Fig. 7). Accordingly, the concentration of 0.2 mM was determined as the optimum concentration to use EMS as the positive control in an alkaline comet assay for short-term exposure (30 min). However, if long-term exposure is preferred (60 min), then 2 mM EMS was determined to be the optimum concentration (Table 2).

**ENU:** A pronounced linear dose dependent increase in the mean tail % intensities were observed across the tested concentrations (0.025–2 mM) at 30 min of exposure duration, as shown in Fig. 5. The mean tail % intensity was significantly higher than the control for 2 mM ENU and the cell viability was higher than 80% at this concentration and exposure duration (Fig. 7). A dose-dependent increase in the mean tail % intensities at an exposure duration of 60 min was not detected. Accordingly, the exposure duration of 30 min and the concentration of 2 mM were determined as the optimum conditions to use ENU as the positive control in alkaline comet assay (Table 2).

**KBrO_3_:** The mean tail intensities were significantly (*P* < 0.05) higher than the control in all tested concentrations at exposure duration of 30 min (Fig. 6). However, a linear increase in tail intensity was not observed across the tested concentrations. The increase in mean tail % intensity stopped at the KBrO_3_ concentration of 500 μM. Under long term exposure (60 min) conditions the mean tail % intensity values were significantly higher than the control for the KBrO_3_ concentrations of 50, 100, 500, and 1000 μM. The cell viability for 2500 μM KBrO_3_ at exposure duration of 30 min was higher than 80%. Accordingly, the concentration of 500 μM was proved as the optimum concentration to use KBrO_3_ as the positive control in alkaline comet assay for short term exposure (30 minutes). However, if long term exposure is preferred (60 minutes) 50 μM KBrO_3_ was the optimum concentration (Table 2).

## Discussion

DNA damage is critical for the maintenance of cellular functions and genetic integrity. It can be caused by a variety of endogenous and exogenous factors. Genotoxicity tests help to identify these damaging agents. The comet assay is one of the most widely used genotoxicity tests, providing rapid visualization of DNA fragmentation in individual cells under alkaline and neutral pH conditions.

The alkaline comet assay is favored for its simplicity and high sensitivity. However, the variability of results between experiments is crucial in assessing the genotoxic potential of a chemical. Using positive controls can help reduce inter-laboratory variability by providing important information about the performance of the comet assay. The OECD guidelines specify preferred positive controls for the in vivo alkaline comet assay, ensuring standardization and minimizing differences between experiments.^15^ Yet, this is not valid for the in vitro alkaline comet assay. As such, positive controls have been used in different concentrations and exposure durations, making it challenging to compare the genotoxic activity of a chemical in the assay when performed in different laboratories.

H_2_O_2_ is the most commonly used positive control in the comet assay, and remarkable differences were observed between different studies in terms of exposure times and concentrations. For example, a systematic review indicated that H_2_O_2_ induced the DNA damage at a concentration of 75 μM and an exposure time of five minutes on ice. Conversely, another study demonstrated that human corneal epithelial cells exhibited heightened sensitivity to DNA damage following a 15- min H_2_O_2_ exposure.^16^^,^^17^ As observed by Rosignoli et al (2001), H_2_O_2_ caused an increase in DNA damage in a concentration-dependent manner, which aligns with the findings of our study. Furthermore, it was observed that this increase in DNA damage diminished at exposure times exceeding 30 min.^18^

Another group of compounds frequently employed in comet assay due to their capacity to induce DNA damage is alkylating agents. Alkylating compounds create the formation of DNA adducts by causing mismatches during DNA replication. Common alkylating agents, including MMS, EMS, and ENU, are utilized as positive controls in the comet assay. Studies have demonstrated that the required exposure time for MMS to induce DNA damage varies across different cell lines but is generally no less than 60 min.^1^^,^^2^^,^^19^ EMS, recommended as a positive control for the in vivo comet assay by the OECD (2016), has been shown to cause a significant increase in tail moment in TX1 tobacco cells after exposure times exceeding 15 min.^20^ Additionally, a study conducted reported that EMS induced DNA damage in CHO-K1 cells at a concentration of 150 μg/mL.^21^ ENU, another alkylating agent identified in the OECD guidelines as a positive control for the in vivo comet assay, has been shown to induce DNA damage in a dose-dependent manner.^22–25^ However, literature data on the use of ENU is more prevalent for in vivo comet assay than for the in vitro.

Etoposide, which differs from hydrogen peroxide and alkylating agents in its effects on DNA, is a cell cycle-specific compound that primarily targets the topoisomerase II enzyme. By forming a ternary complex with DNA, etoposide indirectly causes single and double-chain breaks in DNA.^26^^,^^27^ In various studies evaluating its genotoxic effects, etoposide has been shown to induce DNA damage in Chinese hamster ovary (CHO) cells after a dose-dependent exposure of one hour. Moreover, it has been demonstrated to induce DNA damage in human peripheral blood cells.^26^^,^^28^

KBrO₃, which the European Comet Assay Validation Group (ECVAG) also tested for the enzyme-modified comet assay, induces DNA damage by causing the formation of reactive oxygen products in mammalian cells.^5^^,^^11^^,^^29^ KBrO₃ has been shown to cause DNA damage in human leukocyte cells and rat kidney epithelial cells.^30^^,^^31^ In a study assessing the suitability of KBrO₃ as a positive control for the Fpg-modified comet assay, it was indicated that KBrO₃ exhibited high reproducibility in detecting genotoxic effects. However, notable variability in measured DNA migration was observed, attributed to differences in experimental procedures between laboratories.^11^ A study examining the potential of potassium salts, including KBrO₃, to induce DNA damage found that KBrO₃ caused significant DNA damage and micronucleus formation.^32^ However, data regarding the use of KBrO₃ in the alkaline comet assay are limited, indicating a need for further standardization of potassium bromate to elucidate its role and application in comparison to other genotoxic substances, such as alkylating agents and H_2_O_2_.

The studies mentioned above highlight the lack of a standardized and validated in vitro comet assay, which can lead to variable results obtained from different laboratories and, consequently, varied interpretations of the test results for the same chemical current research is focused on validating the comet assay. In one study involving 14 different laboratories employing their own comet assay protocols, significant inter-laboratory variability was observed in DNA damage results in peripheral blood mononuclear cells exposed to ionizing radiation. However, no significant differences were noted in measurements made by the same method within the same laboratory. It was concluded that the significant differences in DNA damage were attributable to the test protocols used by different laboratories rather than variations in samples obtained from different individuals.^33^ A review of the literature on the variation in test results obtained between laboratories has revealed that the validation process should be conducted at multiple stages of the comet assay procedure, rather than at a single point.^34^^,^^35^ For instance, a study in which the DNA repair capacity was determined by comet assay demonstrated that the discordant results obtained from eight laboratories were attributable to variations in the incubation step.^36^ A study was conducted to ascertain whether the variations in DNA damage observed between laboratories could be reduced by the implementation of the standardised comet assay. The findings indicated that the discrepancies in Fpg-sensitive regions diminished in laboratories utilising the standardised method. However, differences in DNA chain breaks and alkaline-labile regions persisted. This underscores the necessity for further investigation into the utilisation and application of standardised procedures.^37^ The overarching conclusion drawn from these studies is that significant differences exist in the results obtained from unvalidated methods. In this regard, the researchers stated that the data to be obtained from many laboratories are important in standardising and validating the comet assay protocol and that further studies are needed.

Despite comprehensive international collaborative studies, such as the hCOMET project, ECVAG, aimed at reducing inter-laboratory variation, interpreting the comet assay results remains a major concern. This study aims to contribute to this issue by proposing optimization of the utilized positive control’s concentration, exposure duration, and the corresponding DNA damage (e.g. mean tail % intensity).

To minimize variability between different laboratories, it is important to optimize the conditions for the alkaline comet assay based on the positive control-induced DNA migration. This involves optimizing concentrations, exposure durations, and the resulting DNA damage (measured as mean tail % intensity) using positive controls in various cell lines. A specific reference laboratory can determine these optimal parameters, which other laboratories can then use to ensure consistent detection of positive control-mediated DNA damage.

In this study, we have determined the optimal concentrations and exposure durations for several common positive controls (H_2_O_2_, MMS, Etoposide, EMS, ENU, and KBrO_3_) in the alkaline comet assay, along with the corresponding DNA damage levels (e.g. mean tail % intensity), as listed in Table 2. By providing this information, we aimed to guide laboratories to standardize their comet assay procedures and detect consistent DNA damage levels at the same concentrations and exposure durations outlined in our study. Optimizing the concentrations and exposure durations of positive controls based on DNA damage (e.g. mean tail % intensity) could be useful in minimizing the inter-laboratory variations in DNA migration.

## Conclusion

The Comet assay is the gold standard for genotoxicity tests. It is the most widely used method in the world because it is rapid, sensitive, and reliable. The in vivo comet assay is a standardized method included in the OECD guidelines. However, this is not the case in vitro. It is therefore inevitable that test results between laboratories may differ for the same chemical substance.

The use of positive controls is essential for ensuring the accuracy and applicability of a test method. The in vitro comet assay also employs a variety of positive controls. These control compounds ensure the test method is working correctly and reveal the potential genotoxicity profile of the tested chemical compound. It is therefore essential to select an appropriate positive control, determine the concentration of the control to be tested and establish the duration of exposure to the cell line. These factors are crucial for accurately evaluating the method and interpreting the results.

This study aimed to optimize the positive controls used in the in vitro comet assay in terms of concentration and duration in the 3 T3 cell line. These findings are specific to a single cell line. However, making the in vitro comet assay a standard protocol requires consideration of multiple factors. Our study focused on a single cell line. We have determined that 30 and 60 min are the optimal exposure times. Our study provides researchers performing the in vitro comet assay method in the 3 T3 cell line with valuable insight into positive control selection.

To standardize the in vitro alkali comet assay in terms of positive control, studies evaluating different exposure times and concentration values in different cell lines are essential. Researchers using the in vitro comet assay must bring together the findings and share them with the scientific community to accurately evaluate the results of the method.

## References

[1] Bankoglu EE , Schuele C, Stopper H. Cell survival after DNA damage in the comet assay. Arch Toxicol. 2021:95(12):3803–3813. 34609522 10.1007/s00204-021-03164-3 PMC8536587

[2] Bolognesi C , Cirillo S, Chipman JK. Comet assay in ecogenotoxicology: applications in *Mytilus* sp. Mutat Res Genet Toxicol Environ Mutagen. 2019:842:50–59. 31255226 10.1016/j.mrgentox.2019.05.004

[3] Lu Y , Liu Y, Yang C. Evaluating in vitro DNA damage using comet assay. J Vis Exp. 2017:128. 10.3791/56450 PMC5752397 29053680

[4] Gajski G , Ravlić S, Godschalk R, Collins A, Dusinska M, Brunborg G. Application of the comet assay for the evaluation of DNA damage in mature sperm. Mutat Res Rev Mutat Res. 2021:788:108398. 34893163 10.1016/j.mrrev.2021.108398

[5] Møller P . The comet assay: ready for 30 more years. Mutagenesis. 2018:33(1):1–7. 29325088 10.1093/mutage/gex046

[6] Møller P , Azqueta A, Boutet-Robinet E, Koppen G, Bonassi S, Milić M, Gajski G, Costa S, Teixeira JP, Costa Pereira C, et al. Minimum information for reporting on the comet assay (MIRCA): recommendations for describing comet assay procedures and results. Nat Protoc. 2020:15(12):3817–3826. 33106678 10.1038/s41596-020-0398-1 PMC7688437

[7] Petersen EJ , Nguyen A, Brown J, Elliott JT, Clippinger A, Gordon J, Kleinstreuer N, Roesslein M. Characteristics to consider when selecting a positive control material for an in vitro assay. ALTEX. 2021:38(2):365–376. 33637998 10.14573/altex.2102111

[8] Stone V , Johnston H, Schins RP. Development of in vitro systems for nanotoxicology: methodological considerations. Crit Rev Toxicol. 2009:39(7):613–626. 19650720 10.1080/10408440903120975

[9] Milić M , Ceppi M, Bruzzone M, Azqueta A, Brunborg G, Godschalk R, Koppen G, Langie S, Møller P, Teixeira JP, et al. The hCOMET project: international database comparison of results with the comet assay in human biomonitoring. Baseline frequency of DNA damage and effect of main confounders. *Mutat res rev*. Mutat Res. 2021:787:108371. 10.1016/j.mrrev.2021.108371 PMC8525632 34083035

[10] Azqueta A , Ladeira C, Giovannelli L, Boutet-Robinet E, Bonassi S, Neri M, Gajski G, Duthie S, del Bo’ C, Riso P, et al. Application of the comet assay in human biomonitoring: an hCOMET perspective. Mutat Res Rev Mutat Res. 2020:783:108288. 32192646 10.1016/j.mrrev.2019.108288

[11] Møller P , Azqueta A, Collia M, Bakuradze T, Richling E, Bankoglu EE, Stopper H, Bastos VC, Langie SAS, Jensen A, et al. Inter-laboratory variation in measurement of DNA damage by the alkaline comet assay in the hCOMET ring trial. Mutagenesis. 2023:38(5):283–294. 37228081 10.1093/mutage/gead014

[12] Esteves F , Amaro R, Silva S, Sánchez-Flores M, Teixeira JP, Costa C. The impact of comet assay data normalization in human biomonitoring studies outcomes. Toxicol Lett. 2020:332:56–64. 32621954 10.1016/j.toxlet.2020.06.024

[13] Singh NP , McCoy MT, Tice RR, Schneider EL. A simple technique for quantitation of low levels of DNA damage in individual cells. Exp Cell Res. 1988:175(1):184–191. 3345800 10.1016/0014-4827(88)90265-0

[14] Meintières S , Nesslany F, Pallardy M, Marzin D. Detection of ghost cells in the standard alkaline comet assay is not a good measure of apoptosis. Environ Mol Mutagen. 2003:41(4):260–269. 12717781 10.1002/em.10156

[15] OECD. Test No. 489: In vivo mammalian alkaline comet assay. *OECD Guidelines for the Testing of Chemicals, Section 4.* 2016.

[16] Bankoglu EE , Kodandaraman G, Stopper H. A systematic review of the use of the alkaline comet assay for genotoxicity studies in human colon-derived cells. Mutat Res Genet Toxicol Environ Mutagen. 2019:845:402976. 31561903 10.1016/j.mrgentox.2018.10.008

[17] Sakaki H , Kakehi M, Sadamoto K, Nemoto S, Kurata M. In vitro comet assay in cultured human corneal epithelial cells. Fundam Toxicol Sci. 2015:2(4):147–153.

[18] Rosignoli P , Fabiani R, De Bartolomeo A, Spinozzi F, Agea E, Pelli MA, Morozzi G. Protective activity of butyrate on hydrogen peroxide-induced DNA damage in isolated human colonocytes and HT29 tumour cells. Carcinogenesis. 2001:22(10):1675–1680. 11577008 10.1093/carcin/22.10.1675

[19] Ellahueñe MF , Pérez-Alzola LP, Farfán-Urzua M, González-Hormazabal P, Garay M, Olmedo MI, Last JA. Preliminary evaluation of DNA damage related with the smoking habit measured by the comet assay in whole blood cells. Cancer Epidemiol Biomarkers Prev. 2004:13(7):1223–1229. 15247134

[20] Stavreva DA , Ptáček O, Plewa MJ, Gichner T. Single cell gel electrophoresis analysis of genomic damage induced by ethyl methanesulfonate in cultured tobacco cells. Mutat Res Fundam Mol Mech Mutagen. 1998:422(2):323–330. 10.1016/s0027-5107(98)00213-9 9838179

[21] Caffetti JD , Mantovani MS, Pastori MC, Fenocchio AS. First genotoxicity study of Paraná river water from Argentina using cells from the clam *Corbicula fluminea* (Veneroida Corbiculidae) and Chinese hamster (*Cricetulus griseus* Rodentia Cricetidae) K1 cells in the comet assay. Genet Mol Biol. 2008:31(2):561–565.

[22] Fortini P , Raspaglio G, Falchi M, Dogliotti E. Analysis of DNA alkylation damage and repair in mammalian cells by the comet assay. Mutagenesis. 1996:11(2):169–175. 8671734 10.1093/mutage/11.2.169

[23] Stang A , Witte I. Performance of the comet assay in a high-throughput version. Mutat Res Genet Toxicol Environ Mutagen. 2009:675(1–2):5–10. 10.1016/j.mrgentox.2009.01.007 19386240

[24] Sue Marty M , Singh NP, Stebbins KE, Ann Linscombe V, Passage J, Bhaskar GB. Initial insights regarding the role of p53 in maintaining sperm DNA integrity following treatment of mice with ethylnitrosourea or cyclophosphamide. Toxicol Pathol. 2010:38(2):244–257. 20124494 10.1177/0192623309357947

[25] Smith CC , O'Donovan MR, Martin EA. hOGG1 recognizes oxidative damage using the comet assay with greater specificity than FPG or ENDOIII. Mutagenesis. 2006:21(3):185–190. 16597659 10.1093/mutage/gel019

[26] Godard T , Fessard V, Huet S, Mourot A, Deslandes E, Pottier D, Hyrien O, Sichel F, Gauduchon P, Poul JM. Comparative in vitro and in vivo assessment of genotoxic effects of etoposide and chlorothalonil by the comet assay. Mutat Res Genet Toxicol Environ Mutagen. 1999:444(1):103–116. 10.1016/s1383-5718(99)00100-x 10477344

[27] Tammaro M , Barr P, Ricci B, Yan H. Replication-dependent and transcription-dependent mechanisms of DNA double-strand break induction by the topoisomerase 2-targeting drug etoposide. PLoS One. 2013:8(11):e79202. 24244448 10.1371/journal.pone.0079202 PMC3820710

[28] Lebailly P , Vigreux C, Godard T, Sichel F, Bar E, LeTalaer JY, Henry-Amar M, Gauduchon P. Assessment of DNA damage induced in vitro by etoposide and two fungicides (carbendazim and chlorothalonil) in human lymphocytes with the comet assay. Mutat Res Fundam Mol Mech Mutagen. 1997:375(2):205–217. 10.1016/s0027-5107(97)00015-8 9202730

[29] Speit G , Haupter S, Schütz P, Kreis P. Comparative evaluation of the genotoxic properties of potassium bromate and potassium superoxide in V79 Chinese hamster cells. Mutat Res Genet Toxicol Environ Mutagen. 1999:439(2):213–221. 10.1016/s1383-5718(98)00200-9 10023063

[30] Ishidate M Jr , Sofuni T, Yoshikawa K, Hayashi M, Nohmi T, Sawada M, Matsuoka A. Primary mutagenicity screening of food additives currently used in Japan. Food Chem Toxicol. 1984:22(8):623–636. 6381265 10.1016/0278-6915(84)90271-0

[31] Parsons JL , Chipman JK. The role of glutathione in DNA damage by potassium bromate in vitro. Mutagenesis. 2000:15(4):311–316. 10887209 10.1093/mutage/15.4.311

[32] Poul JM , Huet S, Godard T, Sanders P. Lack of genotoxicity of potassium iodate in the alkaline comet assay and in the cytokinesis-block micronucleus test. Comparison to potassium bromate. Food Chem Toxicol. 2004:42(2):203–209. 14667467 10.1016/j.fct.2003.08.018

[33] Ersson C , Møller P, Forchhammer L, Loft S, Azqueta A, Godschalk RW, Van Schooten FJ, Jones GD, Higgins JA, Cooke MS, et al. An ECVAG inter-laboratory validation study of the comet assay: inter-laboratory and intra-laboratory variations of DNA strand breaks and FPG-sensitive sites in human mononuclear cells. Mutagenesis. 2013:28(3):279–286. 23446176 10.1093/mutage/get001

[34] Møller P , Möller L, Godschalk RW, Jones GD. Assessment and reduction of comet assay variation in relation to DNA damage: studies from the European comet assay validation group. Mutagenesis. 2010:25(2):109–111. 20064897 10.1093/mutage/gep067

[35] Møller P . Measurement of oxidatively damaged DNA in mammalian cells using the comet assay: reflections on validity, reliability, and variability. Mutat Res Genet Toxicol Environ Mutagen. 2022:873:503423. 35094807 10.1016/j.mrgentox.2021.503423

[36] Godschalk RW , Ersson C, Riso P, Porrini M, Langie SA, van Schooten FJ, Azqueta A, Collins AR, Jones GD, Kwok RW, et al. DNA-repair measurements by use of the modified comet assay: an inter-laboratory comparison within the European comet assay validation group (ECVAG). Mutat Res Genet Toxicol Environ Mutagen. 2013:757(1):60–67. 10.1016/j.mrgentox.2013.06.020 23830929

[37] Forchhammer L , Ersson C, Loft S, Möller L, Godschalk RW, van Schooten FJ, Jones GD, Higgins JA, Cooke M, Mistry V, et al. Inter-laboratory variation in DNA damage using a standard comet assay protocol. Mutagenesis. 2012:27(6):665–672. 22844078 10.1093/mutage/ges032

